# miR-21, miR-99b and miR-375 combination as predictive response signature for preoperative chemoradiotherapy in rectal cancer

**DOI:** 10.1371/journal.pone.0206542

**Published:** 2018-11-02

**Authors:** Marc Campayo, Alfons Navarro, Jose Carlos Benítez, Sandra Santasusagna, Carme Ferrer, Mariano Monzó, Luis Cirera

**Affiliations:** 1 Department of Medical Oncology, Hospital Universitari Mutua Terrassa, University of Barcelona, Terrassa, Barcelona, Spain; 2 Molecular Oncology and Embryology Laboratory, Human Anatomy Unit, Faculty of Medicine and Health Sciences, University of Barcelona, IDIBAPS, Barcelona, Spain; 3 Department of Pathology, Hospital Universitari Mutua Terrassa, University of Barcelona, Terrassa, Barcelona, Spain; Institut de Pharmacologie Moleculaire et Cellulaire, FRANCE

## Abstract

**Introduction:**

Preoperative chemoradiotherapy (CRT) is a standard treatment for locally advanced rectal cancer patients. Despite the benefits of CRT, its use in non-responder patients can be associated with increased toxicities and surgical resection delay. The identification of CRT response biomarkers, such as microRNAs, could improve the management of these patients. We have studied the microRNA expression in pretreatment endoscopy biopsies from rectal cancer patients treated with CRT to identify potential microRNAs able to predict CRT response and clinical outcome of these patients.

**Material and methods:**

RNA from pretreatment endoscopy biopsies from 96 rectal cancer patients treated with preoperative CRT were studied. Pathological response was graded according to the tumor regression grade (TRG) Dworak classification. In the screening phase, 377 miRNAs were studied in 12 patients with extreme responses (TRG0-1 vs TRG4). The potential role as predictive biomarkers for CRT response, disease-free survival (DFS) and overall survival (OS) of the miRNAs identified in the screening phase were validated in the whole cohort.

**Results:**

In the screening phase, an 8-miRNAs CRT-response signature was identified: let-7b, let-7e, miR-21, miR-99b, miR-183, miR-328, miR-375 and miR-483-5p. In the validation phase, miR-21, miR-99b and miR-375 emerged as CRT response-related miRNAs while miR-328 and let-7e emerged as prognostic markers for DFS and OS. Interestingly, ROC curve analysis showed that the combination of miR-21, miR-99b and miR-375 had the best capacity to distinguish patients with maximum response (TRG4) from others.

**Conclusions:**

miR-21, miR-99b and miR-375 could add valuable information for individualizing treatment in locally advanced rectal cancer patients.

## Introduction

Rectal cancer accounts for approximately one-third of all colorectal tumors, which is one of the leading causes of cancer death in the world[1]. Preoperative chemoradiotherapy (CRT) has been incorporated into common clinical practice for the treatment of locally advanced rectal cancers (T3-T4 or N +). The most frequently used chemotherapy agent is 5-Fluorouracil in combination with concurrent fractionation radiotherapy. Preoperative CRT has multiple advantages, including the higher radiosensitivity of tissues before surgery, the lower rate of toxicities and the higher probability of achieving sphincter preservation due to tumor downstaging[2]. Of note, the rate of pathological response after treatment has been associated with prognosis[2]. Pathological complete response (pCR; ypT0N0), which occurs in 15–25% of the patients, has been linked with lower rates of local recurrences. However, preoperative CRT has not been associated with better disease-free survival (DFS) or overall survival (OS) rates[3, 4]. In clinical practice, there are no validated biomarkers to correctly identify those patients that will not respond, so patient selection remains exclusively a clinically based decision.

MicroRNAs (miRNAs) are short non-coding RNAs that regulate post-transcriptional gene expression by binding primarily to the 3′ untranslated region (UTR) of their target mRNA and repressing its translation[5]. miRNAs are aberrantly expressed and deregulated in cancer[6] where depending on their targets can act either as oncogenes or tumor suppressor genes[7]. Different profiles of miRNAs differentially expressed between rectal cancer and normal rectal tissue have been described[8]. These miRNAs target multiple genes involved in crucial pathways in tumor biology. Moreover, some miRNAs conforming the rectal cancer miRNAome are differentially expressed in colon cancer[9]. Several studies have shown the involvement of miRNAs in resistance to chemotherapy or CRT, both *in vitro* and *in vivo* studies[10–14]. Identifying patients who will not respond to treatment is crucial to avoid unnecessary treatment, potential toxicities and a delay of surgery. In this setting, miRNAs could serve as predictive biomarkers to select the most optimal treatment in each case in order to individualize therapy. Previous studies with a limited number of patients have proposed some miRNAs as predictors of response to preoperative CRT in rectal cancer[15–20].

In the present work, primarily we analyzed the miRNA profile in extreme responder vs non-responder patients to preoperative CRT for rectal cancer treated in our institution. miRNAs differentially expressed between responders and non-responders were studied in a larger series of patients of the same institution and correlated with treatment response and survival.

## Material and methods

### Study population

Ninety-six patients with a diagnosis of rectal adenocarcinoma in a clinical stage II or III (uT3-T4 and/or uN+) and consecutively treated in Hospital Universitari Mutua Terrassa were selected. All patients had received neoadjuvant chemotherapy with 5-Fluouracil 225 mg/m^2^/day x 7 days in continuous infusion and in combination with pelvic locoregional radiotherapy (45–50 Gy). Six to 8 weeks after completion, all patients underwent surgery. All surgical specimens were evaluated and classificated according to TNM 7^th^ edition and pathological response was graded according to the tumor regression grade (TRG) Dworak classification[21]. Approval for the study was obtained from the Institutional Review Board of the Hospital Universitari Mutua Terrassa, Barcelona, Spain.

### RNA extraction

Total RNA was extracted from formalin-fixed paraffin-embedded tumor tissues from pretreatment endoscopy biopsies using RecoverAll Total Nucleic Acid Isolation Kit (Ambion, ThermoFisher Scientific, Waltham, MA, USA) as per the manufacturer's protocol. The concentration of RNA was determined using the NanoDrop ND-1000 Spectrophotometer (NanoDrop Technologies, Wilmington, DE).

### miRNA quantification

In the screening phase, 377 miRNAs were analyzed in 12 selected patients to identify miRNAs associated with response to neoadjuvant treatment. miRNA profiling was performed with TaqMan Array Human MicroRNA A Cards v2.0 (Applied Biosystems, ThermoFisher Scientifics) using pre-amplification as previously described[22]. Expression levels were calculated by the 2^-ΔΔCt^ method. Real-time quantitative PCR reactions were performed on an ABI 7900 HT Sequence Detection System (Applied Biosystems). Normalization was performed with RNU48, based on preliminary analyses comparing the stability of RNU48, RNU44 and MammU6; RNU48 had the lowest variability of expression in the miRNA expression patient data set and was therefore used in this study. All miRNAs that were expressed in less than 10% of samples or with an unreliable quantification were excluded from further analysis, leaving a set of 184 miRNAs.

In the validation phase, selected miRNAs identified in the screening phase were validated in the whole cohort of 96 patients by single Real Time TaqMan MicroRNA Assays in an Applied Biosystems 7500 Sequence Detection System as previously described[23].

### Statistical methods

Data on miRNA expression were analyzed using TIGR Multiexperiment viewer version 4.0 software (The Institute for Genomic Research, and ArrayAssist software, Stratagene, http://www.tm4.org/mev), R software version 3.4 (The R Foundation for Statistical Computing c/o Institute for Statistics and Mathematics, Wirtschaftsuniversität Wien, http://www.r-project.org/) and SPSS v.15.0 (Chicago, IL, USA). Hierarchical clustering was performed using Pearson squared correlation and average linkage. To identify miRNAs with significant differential expression between response groups, two multivariate permutation tests were performed: significance analysis of microarrays (SAM) and Student’s t-test based on multivariate permutation (with random variance model). Differences between miRNAs were considered statistically significant if the P-value was <0.001 (t-test) or false discovery rate <0.1% (SAM). ROC curves were calculated using R package pROC[24]. The multivariate analysis for treatment response was performed by using Binary Logistic regression.

The prognostic impact on DFS and OS, of the identified miRNAs was also evaluated. DFS and OS were defined as the time between surgery and either recurrence or death (DFS) or death from any cause (OS). Optimal cutoffs of miRNA expression data for DFS and OS were assessed by means of maximally selected log-rank statistics[25] using the Maxstat package (R statistical package, v. 2.8.1, Vienna, Austria). DFS and OS were calculated with the Kaplan–Meier method and compared using the log-rank test. All prognostic variables in the univariate analysis with a *P*-value at or below 0.1 were included in the multivariate analysis performed using the stepwise proportional hazard Cox regression model. Statistical significance was set at *P* ≤ 0.05.

## Results

### Patient characteristics

Table 1 shows the main characteristics of the 96 patients included in the study. Sixty-six patients were male and 30 female. Their median age was 66 years (range 38–84 years). Fourteen (15%) patients had clinical stage II and 82 (85%) patients had clinical stage III. 58% of patients received adjuvant therapy after resection. 63% of patients had a downstaging of the disease after treatment, 25% had a pathological complete response (ypT0N0M0) and 27% had a TRG 4.

**Table 1:** Main clinical characteristics of the patients included in the study.

Characteristic			N = 96	DFS	OS
**Sex**	Male		66 (69)	0.619	0.614
	Female		30 (31)		
**Median Age (range)**	66 (38–84)				
	<60		29 (30)	0.154	0.564
	>60		67 (70)		
**Clinical stage pre CRT**	II		14(15)	0.531	0.748
	III		82 (85)		
**Adjuvant therapy**	**No**		40 (42)	0.237	0.346
	**Yes**	5-FU	7 (7)		
		FOLFOX	42 (44)		
		Other	7 (7)		
**ypT**	ypT0		26 (27)	**0.010**	**0.033**
	ypT1-2		23 (24)		
	ypT3-4		47 (49)		
**ypN**	pN0		66 (69)	**0.013**	**0.003**
	pN+		30 (31)		
**Pathological stage**	ypT0N0		24 (25)	**0.047**	**0.044**
	ypI		20 (21)		
	ypII		20 (21)		
	ypIII		32 (33)		
**Downstaging**	No		35 (37)	**0.002**	**0.001**
	Yes		61 (63)		
**Tumor regression grade (TRG)**	0–3		70 (72.9)	0.116	0.149
	4		26 (27.1)		

### Screening phase: Identification of miRNAs related to tumor response

For the primary analyses of miRNA differentially expressed in extreme responders, we selected 6 patients with complete regression (TRG4: no tumor cells detectable in the resected primary tumor after chemoradiotherapy) and 6 patients without response or minor response (TRG 0–1).

**Fig 1:**
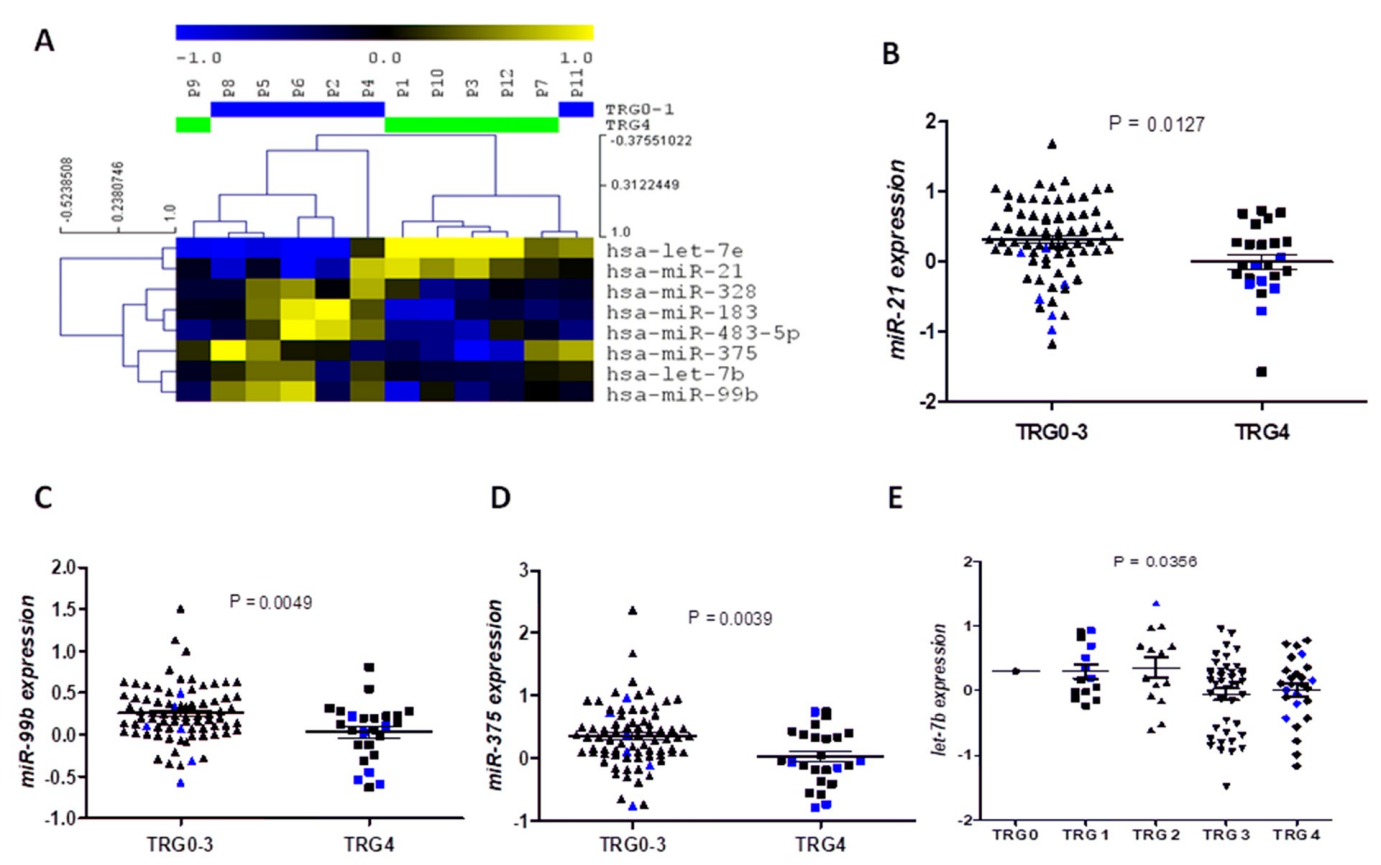
Identification of miRNAs related to treatment response. **A)** Hierarchical cluster analysis showing the 8 miRNAs identified differentially expressed in TRG4 vs TRG0-1 samples in the screening phase. **B)** miR-21, **C)** miR-99b, **D)** miR-375 and **E)** let-7e expression levels in the whole cohort according treatment response (TRG). The black bars in the scatter plots indicate mean ± SEM, and in blue we have highlighted the values from the 12 samples included in the screening phase. TRG 0–3 group is composed of 70 patients, one TRG0, 14 TRG1, 14 TRG2 and 41 TRG3, while the TRG4 group is composed of 26 patients.

Supervised analysis by SAM and t-test of 12 patients revealed 8 common miRNAs differentially expressed in the TRG4 samples compared to the TRG0-1 samples: let-7b, let-7e, miR-21, miR-99b, miR-183, miR-328, miR-375 and miR-483-5p (Fig 1A)

### Validation phase I: Analysis of tumor response-related miRNAs in the whole cohort

The eight miRNAs were studied in the whole cohort (S1 Table and S1 Data) to validate their potential value to predict response to neoadjuvant treatment. Interestingly, those patients who developed complete response (TRG4) had significantly lower miR-21 (p = 0.0127, Fig 1B), miR-99b (p = 0.0049, Fig 1C) and miR-375 expression levels (p = 0.0039, Fig 1D). Moreover, let-7b expression was progressively reduced from TRG0 to TRG4 (Pearson r2 = -0.241, p = 0.014; ANOVA p = 0.0356, Fig 1E). No significant differences were observed for the other miRNAs.

**Fig 2:**
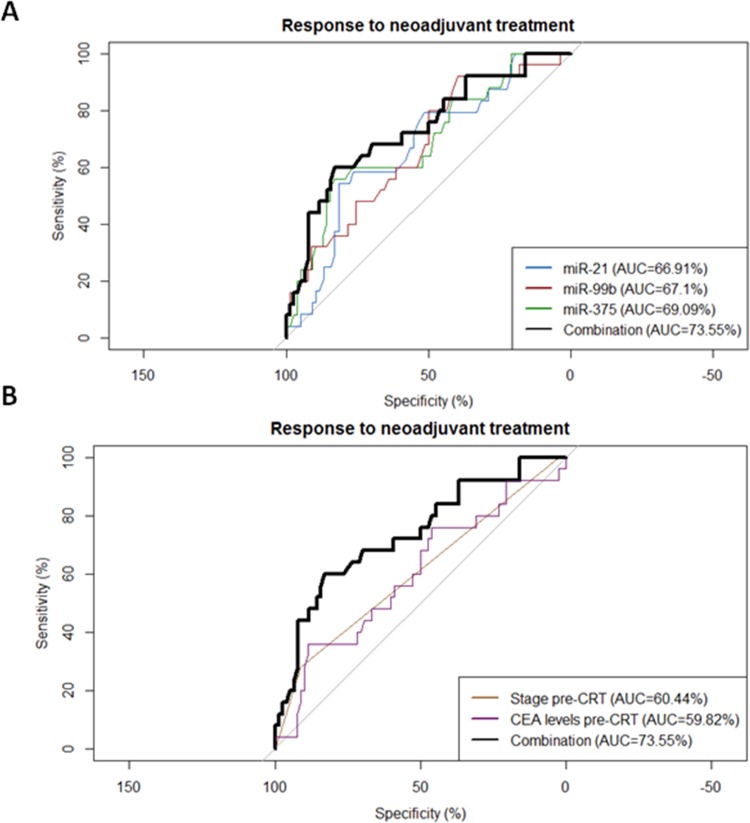
**(A)** ROC curve analyses to evaluate the potential utility of miR-21, miR-99b, miR-375 and their combination to distinguish patients with maximum response to neoadjuvant treatment (TRG4) from others. **(B)** ROC curve analysis comparing the miRNA combination with pre-CRT clinical stage and CEA levels.

Receiver operating characteristic (ROC) curves were generated to investigate the potential utility of miR-21, miR-99b and miR-375 as predictive biomarkers of response to neoadjuvant treatment. The area under the curve (AUC) value for miR-21, miR-99b and miR-375 was 0.6691 (95% confidence interval (CI) = 0.55–0.79; p = 0.012), 0.671 (95% CI = 0.55–0.79; p = 0.01) and 0.691 (95% CI = 0.57–0.81; p = 0.0042) respectively (Fig 2). In the optimum truncation point the sensitivity and specificity was 58.3% and 76.3% for miR-21 (<0.09), 60% and 62% for miR-99b (<0.15) and 60% and 76.6% for miR-375 (<0.07).

Interestingly, the combination of the 3 miRNAs (sum of their expression) improved the discrimination with an AUC value of 0.736 (95% confidence interval (95% CI = 0.62–0.85; p = 0.00043) with 60% sensitivity and 82.9% specificity to distinguish patients with maximum response (TRG4) from others (Fig 2A). In order to compare the impact of the miRNA combination with other clinical factors, pre-CRT clinical stage and pre-CRT CEA levels were included in the analysis. The miRNA combination was a more reliable predictive biomarker than the clinical factors (Fig 2B).

Finally, we performed a multivariate analysis of response to neoadjuvant treatment including sex, age, stage pre-CRT, CEA levels pre-CRT and the miRNA combination. Only the miRNA combination emerged as an independent marker of neoadjuvant treatment response (odds ratio [OR]: 0.439; 95% CI: 0.242–0.797; p = 0.007).

### Validation phase II: Prognosis impact of the tumor response-related miRNAs

Overall median DFS and median OS were not reached. Overall, mean DFS was 79.8 months (95% CI: 71.5–88.1), and mean OS was 89.7 months (95% CI: 82.9–96.5).

There were no statistical significant differences in DFS or OS regarding sex, age, clinical stage, or administration of adjuvant chemotherapy treatment or the TRG (Table 1). On the other hand, there were significant differences on DFS and OS related to pathological stage (DFS: p = 0.047; OS: p = 0.044) or downstaging with CRT treatment (DFS: p = 0.002; OS: p = 0.001).

**Fig 3:**
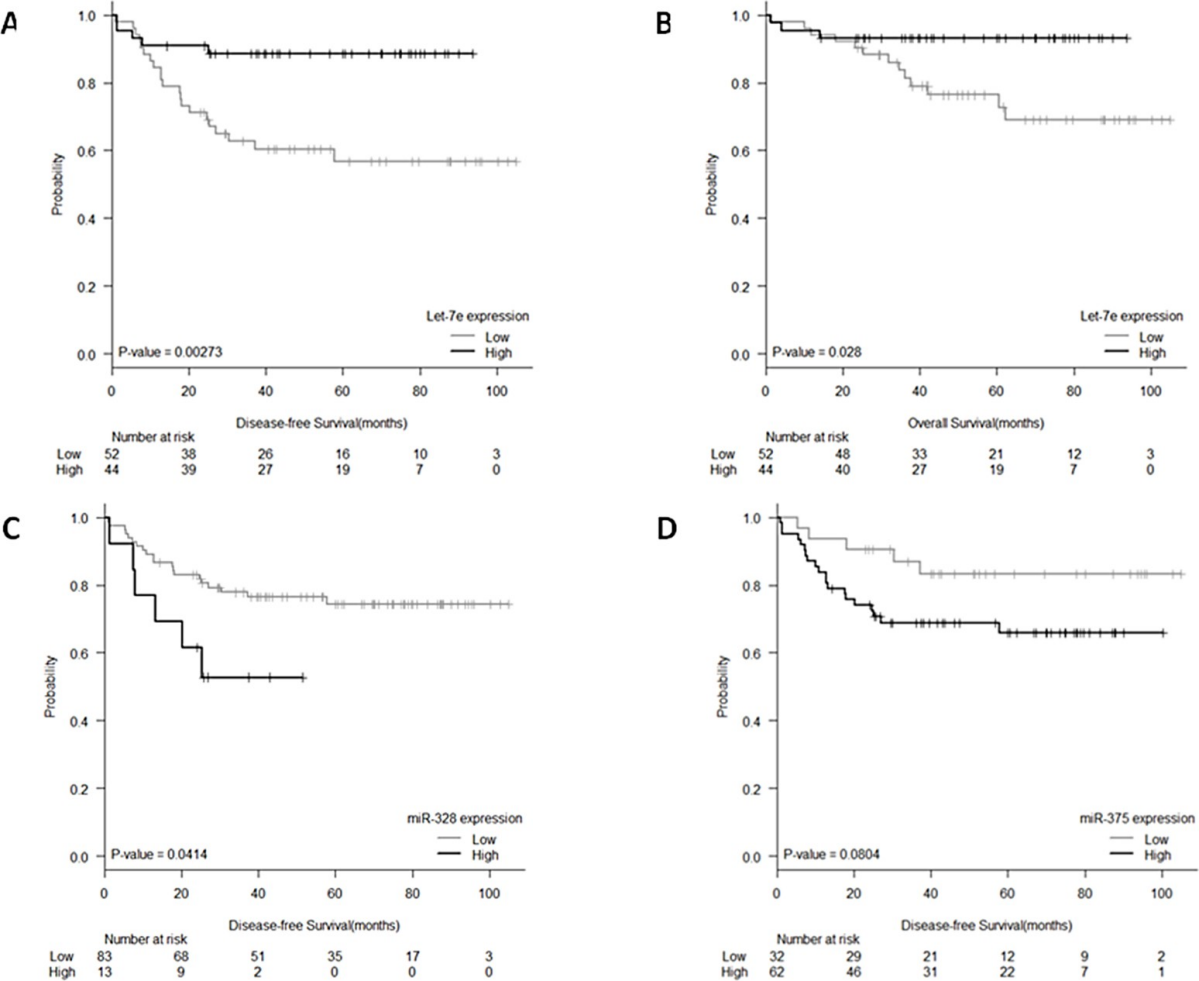
**Kaplan–Meier curves for (A) DFS according to let-7e expression, (B) OS according to let-7e expression, (C) DFS according to miR-328 expression, and (D) DFS according to miR-375 expression**.

**Fig 4:**
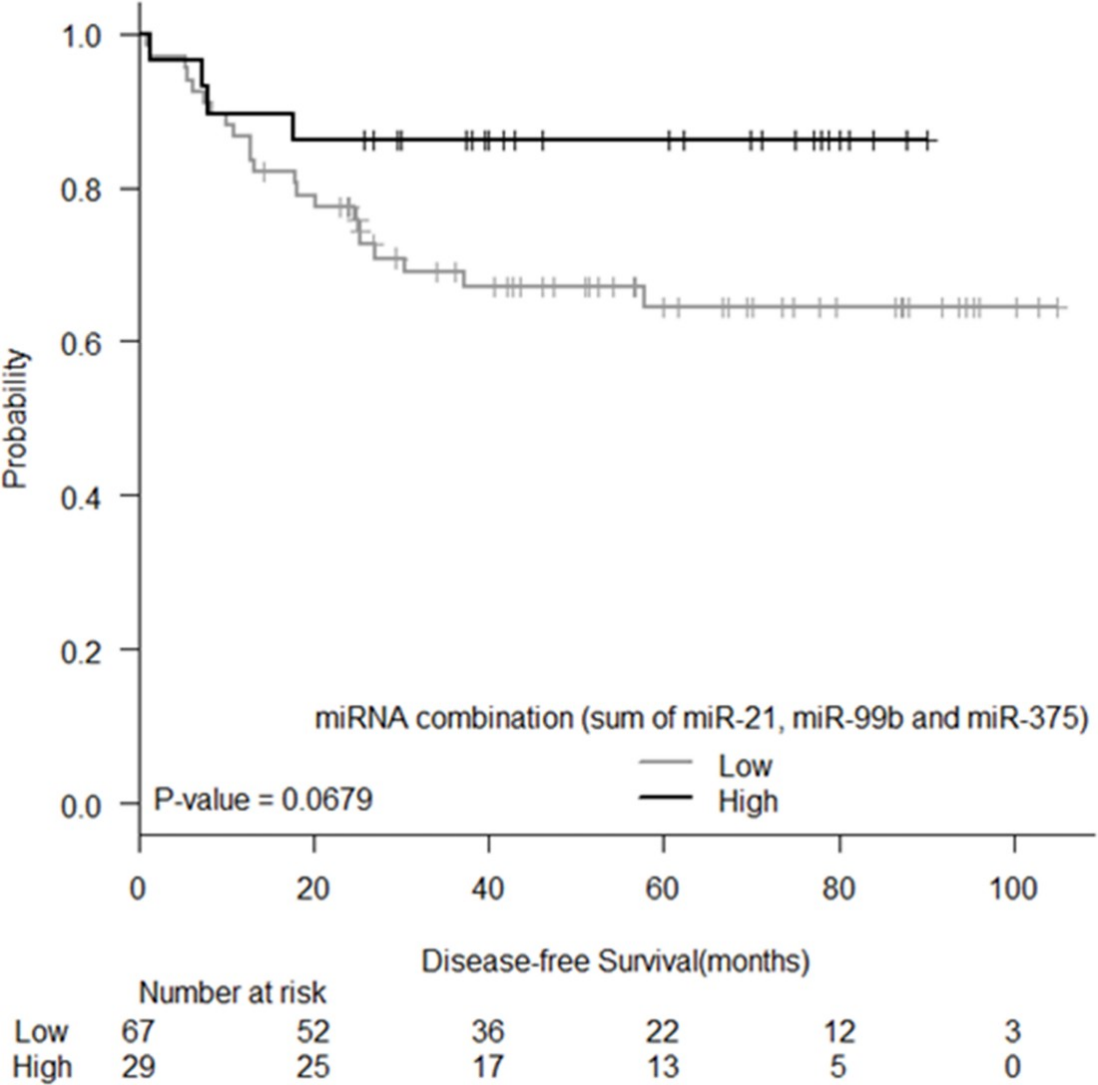
Kaplan-Meier curve for DFS according to the miRNA combination (sum of expression of miR-21, miR-99b and miR-375).

Expression levels of the eight identified miRNAs were correlated with DFS and significant associations were observed for let-7e and miR-328. Mean DFS for patients with low let-7e levels was 68.11 months (95% CI: 55.9–80.3), while it was 83.8 months (95% CI: 75.7–91.9) for those with high levels (P  =  0.003, Fig 3A). Mean DFS for patients with high miR-328 levels was 33.2 months (95% CI: 22.2–44.1), while it was 82.9 months (95% CI: 74.4–91.3) for those with low levels (P  =  0.041, Fig 3C). A trend to significance was also observed for miR-375 where mean DFS for patients with high levels was 71.7 months (95% CI: 61.3–82), while it was 90.8 months (95% CI: 79.5–102.3) for those with low levels (P  =  0.080, Fig 3D). Moreover, we also tested the prognostic impact of the miRNA combination (miR-21, miR-99b and miR-375) and we observed a trend for DFS, where mean DFS for patients with low levels was 74.5 months (95% CI: 64.03–84.9), while it was 78.8 months (95% CI: 68.5–89.1) for those with high levels (P  =  0.068; Fig 4)

Only let-7e was significantly associated with OS. Mean OS for patients with low let-7e levels was 83.1 months (95% CI: 72.9–93.3), while it was 87.7 months (95% CI: 81.2–94.2) for those with high levels (P  =  0.028, Fig 3B).

In the multivariate analyses for DFS (including all factors with univariate p-value ≤0.1: ypN, ypT, pathological stage, downstaging, miR-328 and let-7e), ypT0(OR, 0.278; p = 0.021), high miR-328 expression (OR, 4.340; p  =  0.004) and low let-7e (OR, 6.196; p  =  0.001) emerged as independent prognostic markers for DFS, while downstaging (OR, 0.182; P < 0.003), high miR-328 (OR, 3.905; P  =  0.047) and low let-7e expression (OR, 3.678; P  =  0.044) were independent markers for OS (Table 2).

**Table 2:** Multivariate analysis for DFS and OS.

**Disease-Free Survival**	**Odds Ratio (95% CI)**	**P-value**
ypN0	1.668 (0.458–6.069)	0.438
**ypT0**	**0.278 (0.094–0.826)**	**0.021**
Pathological stage I	0.655 (0.062–6.951)	0.725
Downstaging	0.556 (0.209–1.480)	0.240
**High miR-328**	**4.340 (1.595–11.811)**	**0.004**
**Low let-7e**	**6.196 (2.166–17.722)**	**0.001**
**Overall Survival**	**Odds Ratio (95% CI)**	**P-value**
ypN0	1.104 (0.233–5.236)	0.900
ypT0	0.606 (0.090–4.072)	0.607
Pathological stage I	0.611 (0.025–14.877)	0.762
**Downstaging**	**0.182 (0.058–0.571)**	**0.003**
**High miR-328**	**3.905 (1.017–14.998)**	**0.047**
**Low let-7e**	**3.678 (1.038–13.038)**	**0.044**

## Discussion

Preoperative CRT is a standard treatment for patients with locally advanced rectal cancer and its use is widespread in general clinical practice. However, about 20–40% of the patients do not respond to this neoadjuvant treatment[2]. Patients with a locally advanced rectal cancer are commonly explored with a rectal endoscopy, providing sufficient tissue for diagnosis and for molecular analyses before the start of the treatment. Despite sample availability for molecular analysis, currently there are no clinically validated biomarkers to correctly identify those patients that will not respond. Recent studies have shown an association between pretreatment serum carcinoembryonic antigen (CEA) levels with pCR and survival[26, 27]. Despite this, patient selection is still based on clinical stage and many patients are treated with potential toxic treatments and the definitive surgical resection is delayed [28].

In our present work, we primarily found an eight-miRNA signature that characterize extreme responders (TRG4) using a screening group of patients with well characterized responses (6 TRG0-1 patients vs 6 TRG4 patients). The validation of the eight-miRNA signature in the whole cohort of 96 patients confirmed miR-21, miR-99b, miR-375 and let-7b as preoperative CRT response-related miRNAs. miR-21, miR-99b and miR-375 where significantly downregulated in TRG4 patients vs TRG0-3, while let-7b levels slowly decrease as TRG group increase, with its lower levels in the TRG4 group of patients. Since we were interested in identifying extreme responder patients, we focused in miR-21, miR-99b and miR-375. The ROC curve analysis indicated that these 3 miRNAs were able to predict preoperative CRT response. Interestingly, the combination of them, expressed as the sum of their values, had the best AUC value with a 60% sensitivity and 82.9% specificity to distinguish patients with maximum response from others.

In line with our results, miR-21 overexpression was correlated with non-complete response in a study with 76 rectal adenocarcinomas biopsies before preoperative CRT(RT+5-FU)[29]. However, other studies reported the contrary, that miR-21 overexpression correlated with complete tumor regression[30] [31]. In Eriksen *et al*. study[31], the one with a great cohort, miR-21 was downregulated in responder patients in the test cohort (n = 55, p = 0.062) while was upregulated in responders in the validation cohort (n = 130, p = 0.035). This indicates that the role of miR-21 in preoperative CRT response need to be further studied. In line with our results, *in vitro* studies showed that miR-21 have been found to be overexpressed in 5-FU-resistant colorectal cancer cell lines[32].

miR-99b have been reported upregulated after 5-FU treatment and have predicted targets involved in regulation of autophagy in HT-29 cells[33]. Autophagy has been increasingly recognized as a prosurvival cellular response activated in times of stress during chemotherapy[34] and can promote chemotherapy resistance. miR-99b targets include mTOR signaling pathway which inhibition has been previously related in breast cancer cells with an increase in autophagy[35].

miR-375 overexpression have been previously related to resistance to ionizing radiation and etoposide treatment in gastric cancer cell lines through p53 regulation[36] and elevated miR-375 has been linked to recurrent gastric cancer[37]. In addition, in cervical cancer cell lines overexpression of miR-375 have been related to paclitaxel resistance[38]. In colon cancer, miR-375 have been related to cetuximab resistance through regulation of the AKT pathway by targeting the tumor suppressor gene PHLPP1[39]. Recently, Conde-Muiño et al. described that miR-375 trough regulation of cMyc played a role in chemoresistance to neoadjuvant treatment in locally advanced rectal cancer[40]. All these results are in line with our observation that non-responder patients present high levels of miR-375.

Previous works with lower number of patients have described other miRNAs correlated with response to CRT treatment in rectal cancer that we have not identified in our study: overexpression of miR-31 have been correlated with poor response and worse OS[41]; miR-622 and miR-630 had a 100% sensitivity and specificity in selecting patients with maximum response in a study with 38 patients treated with Capecitabine and Oxaliplatin and radiotherapy[15].

In addition, we studied the potential role of the 8 miRNAs found in the screening phase with the clinical outcome of the patients. In this setting, we found significant differences on DFS regarding expression of let-7e and miR-328 and significant differences on OS depending of expression of let-7e. Moreover, patients with low levels of miR-375 (that correlated with better response) had a trend to better DFS. Interestingly, let-7e and miR-328 emerged as independent prognostic factors for DFS and OS in the multivariate analyses. Patients with high levels of miR-328 and those patients with low levels of let-7e had shorter DFS and OS.

High expression levels of let-7e have been previously correlated with better response to CRT[18]. This result is in line with the results obtained in our screening phase, where we observed that responder patients (TRG4) had higher let-7e levels. Despite we could not validate this result in the whole cohort for treatment response; let-7e emerged as an independent biomarker for DFS and OS. The members of let-7 family have been previously associated with patient outcome in multiple tumors including colorectal cancer[42].

In summary, in the present work we have identified a miRNA signature involved in preoperative CRT response and outcome of rectal cancer patients. Although the present study included a relatively large series with a homogeneous population and treatment; the presented results have to be taken with caution since it is a retrospective study and need to be confirmed in a prospective study. However, miRNAs identified in the present study could be a valuable additional tool for individualizing treatment in locally advanced rectal cancer patients.

## Supporting information

S1 Table Complete analysis results for the eight miRNAs identified in the screening phase according to TRG0-3 vs TRG4 groups. Mean, median, IQR, and non-normalized Ct values. Mean and range are summarized for each group.
(DOCX)

S1 Data Table with the 8 miRNA CT and normalization controls for the full cohort. (XLSX)
