# Supplementary material for: miR-21, miR-99b and miR-375 combination as predictive response signature for preoperative chemoradiotherapy in rectal cancer
Source: PLoS One. 2018 Nov 2;13(11):e0206542. doi: 10.1371/journal.pone.0206542 (PMC6214543; doi:10.1371/journal.pone.0206542)
Supplement: S1 Table — Mean, median, IQR, and non-normalized Ct values. Mean and range are summarized for each group. (DOCX) [file pone.0206542.s001.docx]

**Supplementary Table 1.** Complete analysis results for the eight miRNAs identified in the screening phase according to TRG0-3 vs TRG4 groups. Mean, median, IQR, and non-normalized Ct values. Mean and range are summarized for each group.

| **miRNA** | **Mean** | | **Median** | | **IQR** | | **Non-normalized Ct values – mean (range)** | |
| --- | --- | --- | --- | --- | --- | --- | --- | --- |
|  | **TRG0-3** | **TRG4** | **TRG0-3** | **TRG4** | **TRG0-3** | **TRG4** | **TRG0-3** | **TRG4** |
| **let-7b** | 0.1021 | 0.0105 | 0.1614 | 0.1013 | 0.70 | 0.58 | 26.3 (23.6-29.9) | 26.4 (23.1-29.8) |
| **let-7e** | 0.1402 | 0.0286 | 0.1479 | 0.1178 | 0.49 | 0.46 | 25 (22-31.8) | 25.1 (22.9-28.8) |
| **miR-21** | 0.3183 | 0.0033 | 0.3100 | -0.0500 | 0.64 | 0.59 | 22.8 (19.1-27.6) | 23.6 (20.6-28.2) |
| **miR-99b** | 0.2568 | 0.0328 | 0.2200 | 0.1000 | 0.39 | 0.38 | 28.8 (26.1-32.3) | 29.4 (26.6-34.3) |
| **miR-183** | 0.0096 | 0.0045 | 0.0000 | 0.0000 | 0.04 | 0.25 | 32.6 (32.4-37.2) | 32.9 (30.3-35.6) |
| **miR-328** | -0.0042 | -0.0259 | -0.0662 | -0.1007 | 0.59 | 0.39 | 32.5 (29.2-36) | 32.2 (30.3-34) |
| **miR-375** | 0.3606 | 0.0272 | 0.3300 | -0.0400 | 0.47 | 0.62 | 25.6 (22.7-29.3) | 26.3 (24.1-29.3) |
| **miR-483-5p** | 0.2676 | 0.0571 | 0.1064 | -0.0410 | 0.77 | 0.73 | 30.4 (25.5-33.1) | 30.9 (27.5-34.3) |
